# The Extracytoplasmic Function Sigma Factor SigV Plays a Key Role in the Original Model of Lysozyme Resistance and Virulence of *Enterococcus faecalis*

**DOI:** 10.1371/journal.pone.0009658

**Published:** 2010-03-11

**Authors:** André Le Jeune, Riccardo Torelli, Maurizio Sanguinetti, Jean-Christophe Giard, Axel Hartke, Yanick Auffray, Abdellah Benachour

**Affiliations:** 1 Laboratoire de Microbiologie de l'Environnement EA956 USC INRA 2017, Université de Caen Basse-Normandie, Caen, France; 2 Institute of Microbiology, Catholic University of Sacred Heart, Rome, Italy; University of Hyderabad, India

## Abstract

**Background:**

*Enterococcus faecalis* is one of the leading agents of nosocomial infections. To cause diseases, pathogens or opportunistic bacteria have to adapt and survive to the defense systems encountered in the host. One of the most important compounds of the host innate defense response against invading microorganisms is lysozyme. It is found in a wide variety of body fluids, as well as in cells of the innate immune system. Lysozyme could act either as a muramidase and/or as a cationic antimicrobial peptide. Like *Staphylococcus aureus*, *E. faecalis* is one of the few bacteria that are completely lysozyme resistant.

**Results:**

This study revealed that *oatA* (*O*-acetyl transferase) and *dlt* (D-Alanylation of lipoteicoic acids) genes contribute only partly to the lysozyme resistance of *E. faecalis* and that a specific transcriptional regulator, the extracytoplasmic function SigV sigma factor plays a key role in this event. Indeed, the *sigV* single mutant is as sensitive as the *oatA/dltA* double mutant, and the *sigV/oatA/dltA* triple mutant displays the highest level of lysozyme sensitivity suggesting synergistic effects of these genes. In *S. aureus*, mutation of both *oatA* and *dlt* genes abolishes completely the lysozyme resistance, whereas this is not the case in *E. faecalis*. Interestingly SigV does not control neither *oatA* nor *dlt* genes. Moreover, the *sigV* mutants clearly showed a reduced capacity to colonize host tissues, as they are significantly less recovered than the parental JH2-2 strain from organs of mice subjected to intravenous or urinary tract infections.

**Conclusions:**

This work led to the discovery of an original model of lysozyme resistance mechanism which is obviously more complex than those described for other Gram positive pathogens. Moreover, our data provide evidences for a direct link between lysozyme resistance and virulence of *E. faecalis.*

## Introduction

*Enterococcus faecalis* is a natural member of the gastrointestinal tract of humans and animals. This ubiquitous Gram positive bacterium is abundant in wastewater, on plants, and disseminated in various ecological niches where fecal contamination occurs [1], [2]. It is also an important microorganism of dairy products and some strains are even used as probiotics [3].

While *E. faecalis* is versatile and well suited to survive in hostile environments, under most circumstances, it does not cause any harm to the host. However, on some occasions, the commensal relationship with the host is disrupted leading *E. faecalis* to cause serious diseases [4]. Indeed, *E. faecalis* has emerged as an important opportunistic pathogen, and one of the major causes of nosocomial infections such as urinary tract infection, endocarditis, and surgical wound [5]. In addition, due to its innate and acquired resistances to several antibiotics including in some cases vancomycin, conventional therapies are insufficient to threat *E. faecalis* serious infections [6]. Although several genes for virulence factors in *E. faecalis* have been characterized, and their effects have been demonstrated in animal models or cultured cells [7], the mechanisms by which this peaceful commensal became a life-threatening pathogen are not well understood [4].

As for other pathogens, in order to survive and colonize a given host, *E. faecalis* must successfully overcome specific and non specific host defense mechanisms. One of the most important and widespread compounds of this defense system against invading micro-organisms is lysozyme. This enzyme is found in a wide variety of body fluids, such as tears, breast milk, respiratory and saliva secretions, as well as in cells of the innate immune system, including neutrophils, monocytes, macrophages, and epithelial cells [8]–[10]. Lysozyme acts on bacteria by hydrolyzing the β-1,4 glycosidic bonds between *N*-acetylmuramic acid (MurNAC) and *N*-acetylglucosamine (GlucNAC), resulting in the degradation of the peptidoglycan (PG), and subsequent cell lysis. It has also a cationic anti-microbial peptide (CAMP) function that leads to bacterial death likely through the destabilization of the cytoplasmic membrane [11]–[13].

In contrast to the majority of bacteria, and like some important human pathogens such as *Staphylococcus aureus* or *Neisseria gonorrhoeae*, *E. faecalis* is completely resistant to lysozyme. Three main mechanisms involved in this resistance have been well described for different species of bacteria. Two of them counteract antibacterial activity of lysozyme: i) the modification of different sites of the PG structure by two kinds of enzymes such as the *N*-acetylglucosamine deacetylase (PgdA) of *Streptococcus pneumoniae* [14], or the peptidoglycan-specific *O*-acetyltransferase (OatA) of *S. aureus* [15] prevents the binding of lysozyme to its substrate and contributes to the muramidase resistance; ii) the modification of the net negative charge of the bacterial cell surface by adding positively charged residues (D-alanine esterification through *dlt* genes) to teichoic and lipoteichoic acids helps bacteria to avoid being killed by antimicrobial peptides or CAMP activity of lysozyme [13], [16]. The third mechanism to evade the killing action of lysozyme consists on the production of lysozyme inhibitors such as the streptococcal inhibitor of complement (SIC) in streptococci [17], the inhibitor of vertebrate lysozyme (Ivy) in *E. coli* [18], and the periplasmic (or membrane) lysozyme inhibitor of c-type lysozyme (named PliC or MliC, in *Salmonella enteritidis* and *E. coli*, respectively) [19], which have protective function.

The availability of *E. faecalis* genome sequence [20] provides a tool for the identification of new virulence factors which could help understanding pathogenicity mechanisms and regulatory components, such as alternative sigma factors, that could be involved in their infection processes. Bacterial sigma factors are a class of proteins constituting essential dissociable subunits of RNA polymerase to direct the initiation of the transcription from specific promoter sequences [21], [22]. In addition to the “housekeeping” sigma factor (i.e. RpoD or σ^70^ of *E. coli*), most bacteria have multiple alternative sigma factors that they use to coordinately regulate the expression of genes whose products are involved in diverse functions, such as stress responses, iron uptake, virulence, morphological development, and chemotaxis [23], [24]. Like those of *Streptococcus pyogenes*, *S. pneumoniae*, and *Lactococcus lactis*, the *E. faecalis* genome lacks a general stress response regulator homologous to σ^B^ of *Bacillus subtilis* [25]. However, in a previous work, we have shown that the extracytoplasmic function (ECF) sigma factor SigV contributes to survival following heat, acid and ethanol treatments, and is also likely involved in lysozyme resistance of *E. faecalis* [26]. In *E. faecalis*, this lysozyme resistance was only in part related to *oatA* gene [27].

This study is dedicated to the continuum of the story of *E. faecalis* lysozyme resistance. Based on *in silico* analyses and on previous studies [13], [16], [26], [27], we analyzed in depth the role of some genes involved (*oatA*) or suspected to be involved (*sigV*, *dlt* and *mprF*) in the *E. faecalis* lysozyme resistance. We constructed their corresponding mutants and analyzed their behavior comparatively to the parental strain. Thus, we showed that in addition to the pleiotrophic effects displayed by SigV, it also contributes importantly to the lysozyme resistance and virulence of *E. faecalis*.

## Results

### Identification of genes involved or suspected to be involved in lysozyme resistance

Based on preliminary results, we have already suggested that *sigV* (*EF3180*) gene encoding an ECF sigma factor is likely involved in lysozyme resistance of *E. faecalis* [26]. A further study revealed that among the two main genes *pgdA*-like (*EF1843*) and *oatA* (*EF0783*) able to confer this lysozyme resistance through the modification of different sites of the PG structure, only *oatA* has a significant role in *E. faecalis* [27].

**Figure 1:**
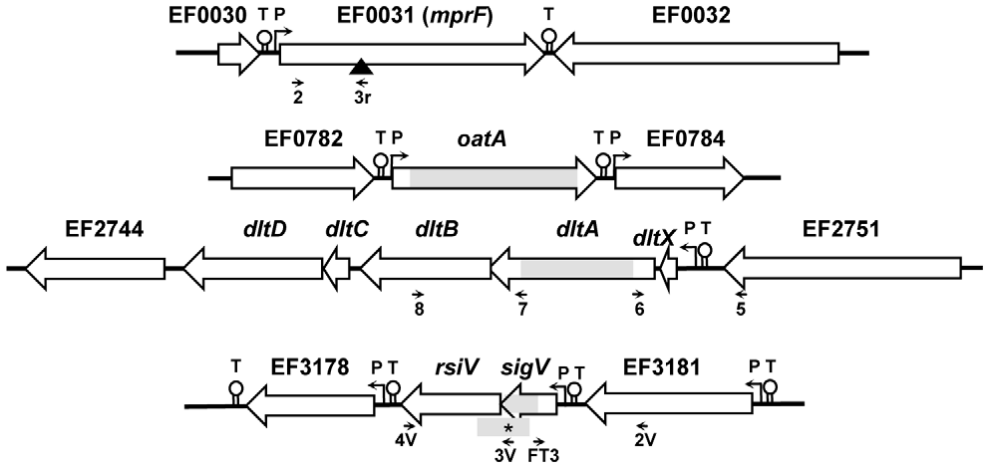
Structural organization of *sigV, oatA, dltA* and *mprF* loci. Large arrows correspond to the indicated genes. Grey areas show the deleted region by double cross over event and that of *sigV* locus (harbouring an asterisk) represents the deletion carried out in SAS mutant strain. The solid triangle positioned on EF0031 shows the insertion site of the integrative pUCB300-*mprF* recombinant plasmid generating the *mprF* mutant. The putative promoters and terminators are indicated with P and T letters, respectively. Primers used to construct the different mutants are indicated by black arrows, and listed in Table 4.

In the context of cell envelope net charge that may have a role in lysozyme resistance, we included the analysis of the *dlt* operon (D-alanylation of lipoteichoic acids) for which evidence in lysozyme resistance mechanism was established in *S. aureus* [13]. The genetic organization of the *dlt* operon of *E. faecalis* as reported by Fabretti *et al*., [28] is composed by 4 genes *dltA*, *B*, *C*, and *D* (*EF2749*, *EF2748*, *EF2747*, and *EF2746*, respectively) where *dltA* encodes a putative D-alanine-activating enzyme. However, a careful analysis of the genome sequence of *E. faecalis* V583 strain revealed an additional gene *dltX* (*EF2750*), upstream of *dltA* which likely belongs to the *dlt* operon (Fig. 1). This gene encodes a putative short protein (49 amino acid residues), homologous to that described for *S. aureus* and highly conserved among Gram positive bacteria [29].

The *in silico* analysis of the V583 genome sequence has also revealed the presence of *EF0031* gene, whose product shares 24% identity and 45% homology with MprF (multiple peptide resistance factor) of *S. aureus*. This protein contributes to the modification of the membrane net charge by incorporation of L-lysine to phosphatidylglycerol. This reduces attractive electrostatic interaction and thereby, the binding of antimicrobial peptides by bacteria [30]. In addition, Blast searches using different databanks did not lead to identification of any obvious *E. faecalis* homologue to lysozyme inhibitors such as Ivy, Sic, MliC or PliC.

Thus, for this work, we focused on the analysis individually or in combination of *sigV*, *oatA*, *dltA*, and *mprF* genes whose structural organizations are represented in Fig. 1.

### Sensitivity to lysozyme

**Figure 2:**
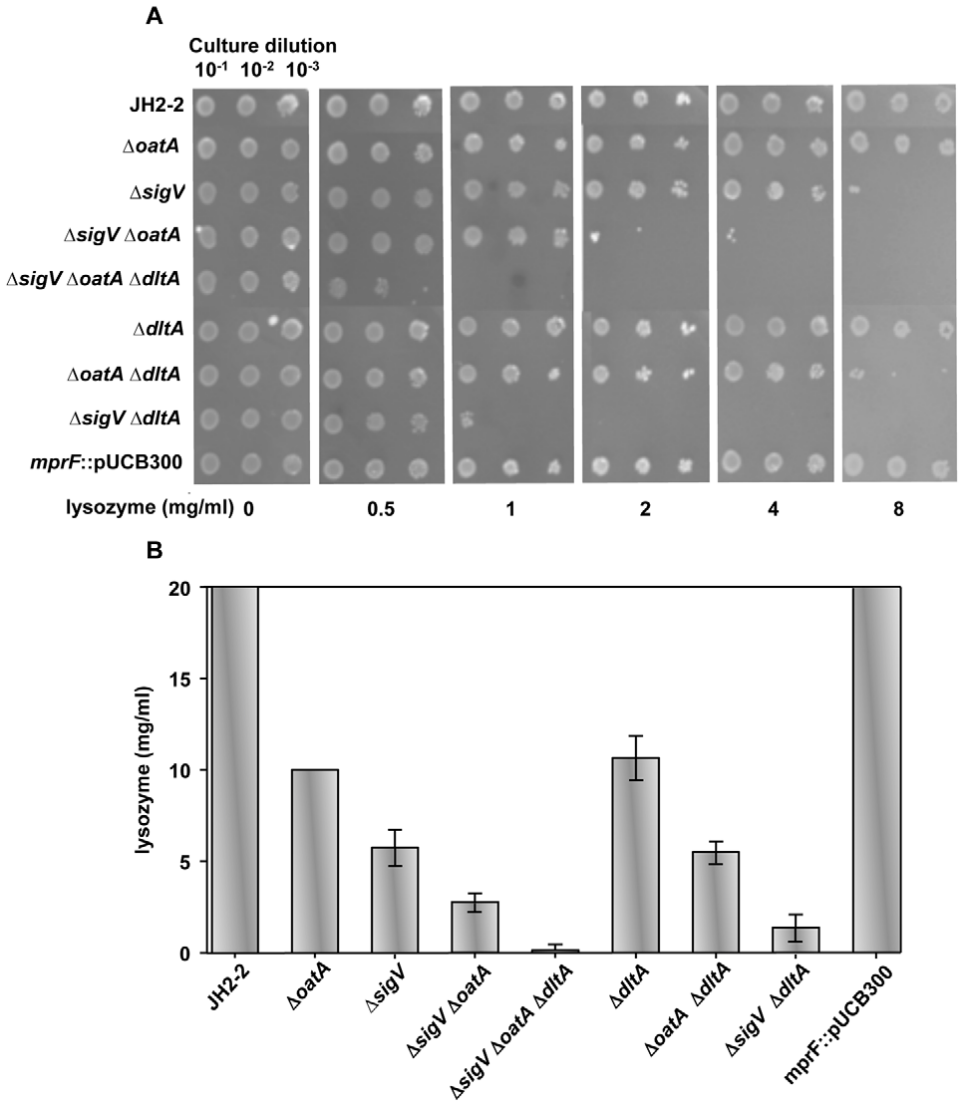
Susceptibility to lysozyme. The lysozyme sensitivity of *E. faecalis* JH2-2 and its derivative mutants was tested on LB agar medium. Overnight cultures were adjusted to OD_600_ = 1 in physiological water, and diluted up to 10^−3^. Equal volume of each of the 10^−1^, 10^−2^, and 10^−3^ dilutions are plated on LB solid medium containing large scale range of lysozyme concentrations. (**A**) Photographs of LB plates after 48 hours incubation under the indicated lysozyme concentrations. (**B**) The overall results of the lysozyme sensitivity experiments are summarized in this histogram.

Prior to lysozyme treatment, the parental *E. faecalis* JH2-2 strain and its isogenic derivative mutants were tested for their growth behavior on broth or solid media. Monitoring growth kinetics on GM17 broth revealed no significant difference for all mutants relatively to the parental JH2-2 strain (data not shown). Plating the strains on LB solid medium revealed also a similar result after 48 hours incubation at 37°C in absence of lysozyme (Fig. 2A).

The parental JH2-2 strain and its derivative mutants were tested for lysozyme sensitivity on LB plates containing large-scale range of lysozyme concentrations (0 to 20 mg/ml). The wild type JH2-2 strain was not affected in its growth at 20 mg/ml of lysozyme and even at higher concentrations (data not shown). Surprisingly, also the *mprF* insertional mutant was as resistant as the JH2-2 parental strain (Fig. 2A). This result showed that *mprF* (suspected to avoid CAMP activity) is not involved in lysozyme resistance mechanism of *E. faecalis*, and was no longer investigated in this work. In contrast, the other mutants tested do not grow at 20 mg/ml of lysozyme. Thus, for the histogram representation of the results (Fig. 2B), this concentration was chosen as the highest limit.

Both *oatA* and *dltA* single mutants were similarly more sensitive to lysozyme than JH2-2 parental strain, but inhibition of growth still needs at high concentration of lysozyme (about 10 mg/ml) (Fig. 2B). This confirms previous results showing that *oatA* is not the main effector of *E. faecalis* lysozyme resistance [27], and that *dltA* is also involved in this mechanism. The *oatA/dltA* double mutant was more lysozyme sensitive (about 5 mg/ml) than each of the single mutants (Fig. 2B). This additive effect of both genes is in agreement with the dual activities (muramidase and CAMP) of lysozyme.

The *sigV* single mutant showed a sensitivity level (5 mg/ml) similar to that of *oatA/dltA* double mutant (Fig. 2A and 2B). Comparatively to *sigV* mutant, *sigV/oatA* and mainly *sigV/dltA* double mutants showed increased sensitivity to lysozyme, and *sigV/oatA/dltA* triple mutant is the most sensitive (starting from 0.5 mg/ml) among all the strains tested (Fig. 2A and 2B). The synergistic effect of *sigV* deficiency in the *oatA/dltA* double mutant highlights the key role of the ECF SigV sigma factor in the lysozyme resistance mechanism of *E. faecalis*.

**Figure 3:**
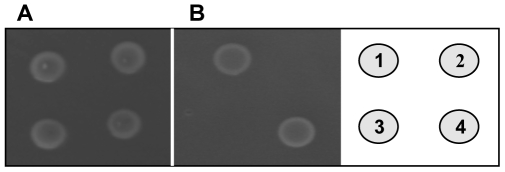
Complementation assay under lysozyme treatment. The parental strain *E. faecalis* JH2-2 (**1**), and its derivative mutants Δ*sigV* (**2**), SAS pMSP3535 (**3**), and SAS pMSP3535-*sigV* (**4**) were grown on LB solid medium without lysozyme (**A**) or supplemented with 20 mg/ml of lysozyme (**B**). Five µl of 10^−2^ dilution of overnight cultures adjusted to OD_600_ = 1 in physiological water were plated on solid medium, incubated at 37°C for 48 hours and photographed.

In order to confirm the specificity of SigV in its involvement in lysozyme resistance, we undertook a complementation assay using JH2-2 parental strain and its derivatives *sigV*, SAS pMSP3535-*sigV* (expressing SigV), SASpMSP3535 (control). Growth on LB solid medium without (Fig. 3, panel A) or with 20 mg/ml of lysozyme (Fig. 3, panel B) showed that SigV was able to restore the lysozyme resistance phenotype even at a concentration of 20 mg/ml (Fig. 3, panel B).

### Are *oatA* and *dltA* genes under the control of SigV?

Regarding the results reported above, we wondered if there is a link at the transcriptional level between *sigV*, *dltA*, and *oatA* genes. Since *EF1843* gene (*pgdA*-like) shares the same promoter sequence than *sigV* [26], we included it as a positive control for RT-qPCR analyses. The results of Table 1 clearly showed that *sigV* and *pgdA*-like are overexpressed when cells were exposed to 3 mg/ml lysozyme treatment for 30 minutes. Indeed, *sigV* and *pgdA*-like were similarly induced in JH2-2 strain since they are 320 fold and 247 fold overexpressed with significant *p* values of 0.001 and 0.027, respectively whereas *dltA* and *oatA* genes can be considered as expressed only at a basal level. Under the same conditions, expression of *pgdA*-like, *dltA*, and *oatA* genes was not significantly influenced by exposition to lysozyme in the *sigV* mutant (Table 1).

**Table 1:** RT-qPCR values under lysozyme (3 mg/ml) treatment.

		RT-qPCR(a)	
Orfs	Gene name	JH2-2	Δ*sigV*
***EF3180***	*sigV*	+320 (p = 0.001)	nd
***EF1843***	*pgdA*-like	+247 (p = 0.027)	−1.21 (p = 0.917)
***EF2749***	*dltA*	+1.83 (p = 0.125)	+1.02 (p = 0.445)
***EF0783***	*oatA*	+2.4 (p = 0.053)	+1.26 (p = 0.263)

(a) Factor of activation or repression determined by RT-qPCR in JH2-2 wild-type strain and *sigV* mutant exposed to 3 mg/ml lysozyme for 30 minutes. Values >2 with *p* value <0.05 are considered as significant. nd, not determined.

In the JH2-2 strain, *sigV* and *rsiV* genes constituting a bicistronic operon are expressed in the same way [26] and the *rsiV* mutant was as resistant as the parental JH2-2 strain (data not shown). Regarding that most ECF sigma factors are auto regulated, we wondered if the overproduction of SigV will lead to consistent overexpression of *sigV* and what will be the incidence on the expression of *pgdA*-like, *dltA*, and *oatA* genes. For this purpose, SigV was overproduced using SAS pMSP3535*-sigV* and SAS pMSP3535 (control) strains under appropriate condition of nisin (0.5 µg/ml) induction and we analyzed the transcription of these genes using RT-qPCR. The results reported in Table 2 revealed that only *sigV* and *pgdA*-like genes are drastically overexpressed (3983 fold and 1992 fold induced, respectively), whereas no enhanced expression of *oatA* and *dltA* genes was observed demonstrating that both genes are not under the control of SigV.

**Table 2:** RT-qPCR values under nisin (0.5 µg/ml) treatment.

Orfs	Gene name	RT-qPCR(a) Reference 23S	
*EF3180*	*sigV*	+3983	+3191
*EF1843*	*pgdA*-like	+1992	+1884
*EF2749*	*dltA*	−1.25	−1.5
*EF0783*	*oatA*	−1.01	+1.1

(a) Factor of activation or repression determined by RT-qPCR when *sigV* is overexpressed in the SAS pMSP3535-*sigV* compared to SAS pMSP3535 (control).

### Is *sigV* involved in the resistance to CAMP activity?

**Figure 4:**
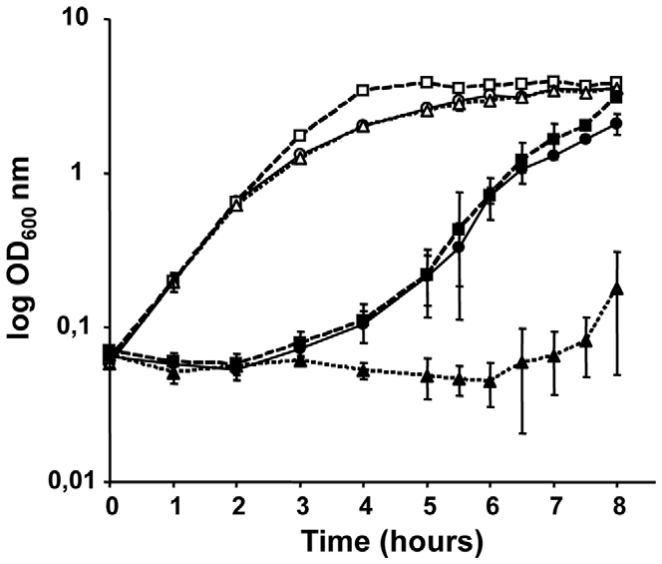
Susceptibility to nisin. The sensitivity to nisin of *E. faecalis* JH2-2 (•) and its derivative mutants Δ*sigV* (▪) and Δ*dltA* (▴) was tested on GM17 medium (open symbols) or on GM17 supplemented with 2 µg/ml of nisin (solid symbols). The kinetic growth was monitored at OD_600_ nm. Mean values of three independent experiments are shown and standard deviations are indicated.

Both lysozyme and nisin possess a cationic domain involved in membrane destabilization. To avoid CAMP activity, bacteria have evolved mechanisms through the reduction of the cell envelope net charge mainly mediated by *dlt* genes as it has been described for *S. aureus, E. faecalis* and many other bacteria [15], [16], [28]. In order to determine whether SigV contributes to the resistance towards CAMP activities, we monitored growth in the absence or presence of nisin (the representative model of CAMPs) of *sigV* and *dltA* (control expected to be affected by nisin activity) single mutants comparatively to *E. faecalis* JH2-2 parental strain. In the absence of nisin, all strains had similar growth on GM17 broth (Fig. 4). In the presence of nisin, the three strains showed a similar prolonged lag phase (3 hours), and subsequently only the *dltA* mutant demonstrated enhanced sensitivity towards nisin (Fig. 4). Interestingly, the *sigV* mutant behaved similarly to the parental JH2-2 strain and was able to grow on GM17 supplemented with 2 µg/ml of nisin after an adaptation period (Fig. 4). These results strongly suggest that SigV ECF sigma factor does not contribute to the resistance towards CAMP activity in the tested conditions.

### Triton X-100-induced autolysis assays

**Figure 5:**
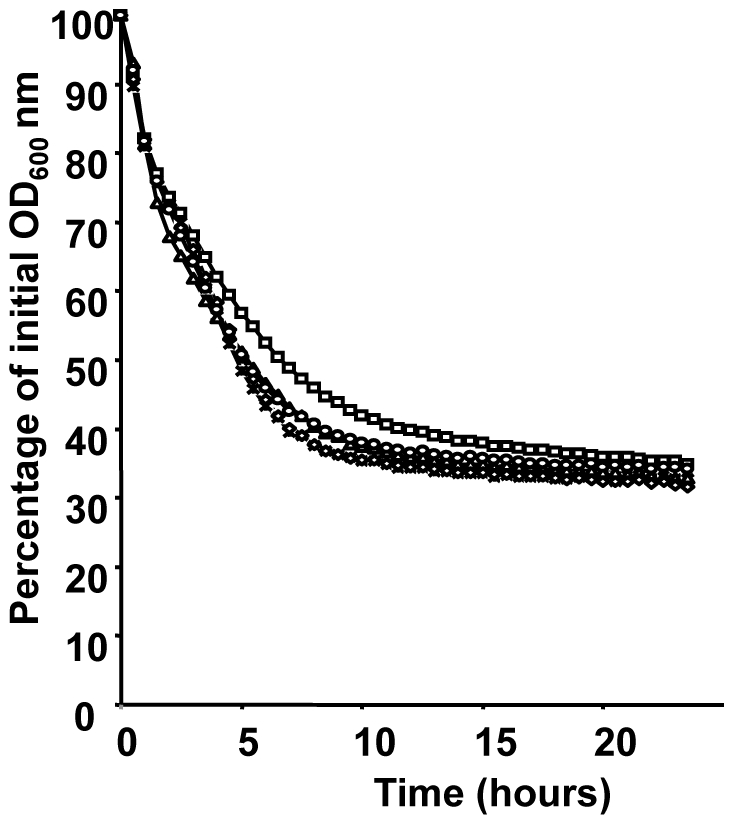
Triton X-100-induced autolysis assays. The autolysis of *E. faecalis* JH2-2 (○) and its derivative mutants Δ*sigV* (□), Δ*dltA* (Δ), Δ*oatA* (◊), and Δ*sigV/oatA/dltA* (*) was carried out in Tris-HCl buffer containing 0.1% of Triton X-100. The autolysis was monitored by measuring the decrease in OD_600_ nm of the cell suspensions every 30 min.

Mechanisms affecting the modification of the membrane net charge or the PG structure play a role in the modulation of autolysins activity and thus, may have an impact on bacterial autolysis [31]–[34]. In this context, we evaluated the effect of autolysis induction by triton-X100 on *oatA*, *sigV*, *dltA* single mutants, *sigV/oatA*/*dltA* triple mutant, and JH2-2 wild-type strain. Over a period of 5 hours, there was a decrease in OD_600_ nm of about 50%, and reaching 65% after overnight incubation for all strains tested towards exposure to 0.05% or 0.1% of Triton X-100 (Fig. 5). This result suggests that *oatA*, *sigV*, and *dltA* genes are not involved in autolysis process as it was already reported for the *dlt* mutant of *E. faecalis* 12030 strain [28].

### SigV is involved in systemic and urinary tract infection models

In order to assess the effects of *oatA*, *dltA*, and/or *sigV* genes deletion on the virulence of *E. faecalis* JH2-2, we compared the fate of the wild-type and its derivative mutant strains in two different infection mouse models.

**Figure 6:**
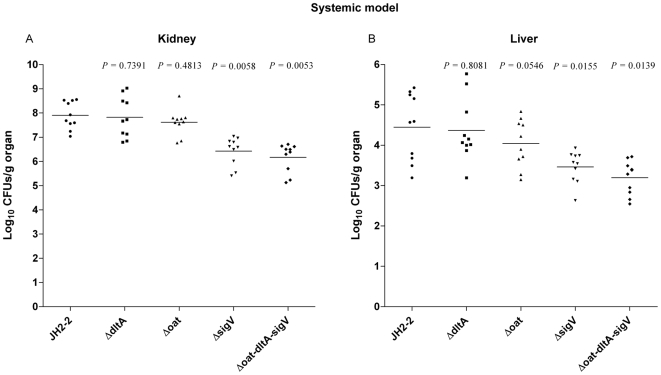
SigV is required for the virulence of *E. faecalis* in a murine systemic model. Enterococcal tissue burdens in kidneys (**A**) and in livers (**B**) from BALB/c mice infected intravenously with 5×10^8^ cells of *E. faecalis* JH2-2 wild-type (•) and its isogenic mutant strains Δ*dltA* (▪), Δ*oat* (▴), Δ*sigV* (▾), and Δ*dltA*-*oat*-*sigV* (♦). Kidney pair and livers homogenates were obtained from groups of 10 mice sacrificed and necropsied at day 7 post-infection. Results, expressed as log_10_ CFUs per gram of tissue, represent values recorded separately for each of the 10 mice. Horizontal bars represent the geometric means. *p* value of less than 0.05 was considered to be significant.

At first, by using a well-established intravenous infection model [35], we compared tissue burdens in kidneys and livers of groups of infected mice. As shown in Fig. 6, only *sigV* and *sigV/oatA*/*dltA* mutants showed statistically significant reductions of tissue burden both in kidneys and livers. In particular, the *sigV* mutant exhibited a reduction of 1.51 logs (*p* = 0.0058) in kidneys and of 1.37 logs (*p* = 0.0155) in livers as compared to the JH2-2 wild-type strain. Similar results were obtained for the triple mutant (−1.77 logs in the kidney, *p* = 0.0053; and −1.60 logs in the liver, *p* = 0.0139), suggesting that *sigV* is involved in *E. faecalis* virulence. However, it is noteworthy that while non significant, OatA seems to display a slight reduction of tissue burdens in livers (−0.40 logs, *p* = 0.0546), following systemic infection in mice as it could be seen in Fig. 6B.

**Figure 7:**
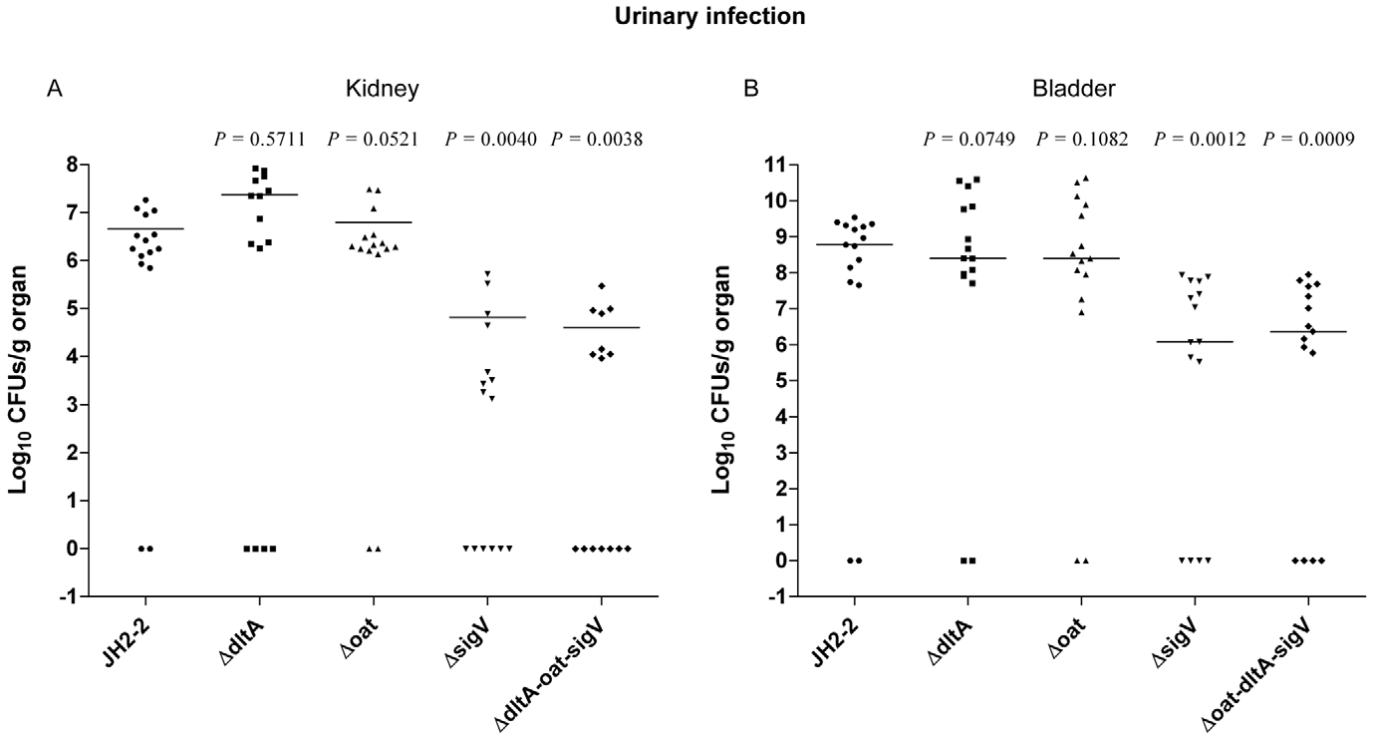
SigV is required for the virulence of *E. faecalis* in a murine UTI model. Enterococcal burdens of kidneys (**A**) and bladders (**B**) of Balb/c mice infected transurethrally with 10^4^ cells of *E. faecalis faecalis* JH2-2 wild-type (•) and its isogenic mutant strains Δ*dltA* (▪), Δ*oat* (▴), Δ*sigV* (▾), and Δ*dltA*-*oat*-*sigV* (♦). Kidney pair and bladders homogenates were obtained from groups of 15 mice that were sacrificed and necropsied 48 h after the transurethral challenge. Results, expressed as log_10_ CFUs per gram of tissue, represent values recorded separately for each mouse. Horizontal bars represent the geometric means. A value of 0 was assigned to uninfected kidneys or bladders. *p* value of less than 0.05 was considered to be significant.

The same strains were also tested in a urinary tract infection (UTI) model as described elsewhere [36]. The resulting ID_50_s showed that only the *sigV* single and triple mutants required 0.82 and 1.03 log_10_ more cells (4.6×10^3^ and 7.4×10^3^, respectively) than did the JH2-2 wild-type strain (6.9×10^2^) to infect 50% of the mice, thereby suggesting that in the UTI model only *sigV* was implicated in *E. faecalis* infection (Fig. 7). Thus, percentages of kidneys of mice infected for all the bacterial inoculates used were 84% for the JH2-2 wild-type strain, 58% for the *sigV* mutant (*p* = 0.0047), and 53% for the triple mutant strain (*p* = 0.0012). Also, the cumulative difference between the bladders infected with the two strains was similar to that of the infected kidneys (71% for the JH2-2 versus 40% for the Δ*sigV*, *p* = 0.0066; and 71% for the JH2-2 versus 34% for the triple mutant, *p* = 0.0015). The Fig. 7 shows the log_10_ CFU recovered from the kidney pairs of mice infected with 10^4^ cells from JH2-2 or each of mutant strains. As for the systemic infection model, only the strains Δ*sigV* and triple mutant exhibited statistically significant reductions (−1.83 logs for the Δ*sigV*, *p* = 0.004; and −2.04 logs for the triple mutant, *p* = 0.0038) in kidneys tissue burden as compared to the wild-type strain (Fig. 7A). Similar results were obtained when analyzing bladders. In fact, in this case also, only *sigV* single and triple mutants showed statistically significant reductions (−2.42 logs, *p* = 0.0012; and −2.32 logs, *p* = 0.0009, respectively) in tissue burdens when compared to the wild-type strain (Fig. 7B).

## Discussion

Environmental adaptation traits and stress resistance mechanisms have been linked to virulence, as bacterial survival in the host is often reliant on these factors. Despite its ability to adapt to many different environmental stresses, *E. faecalis* possesses a moderate number of regulatory genes [37]. Among these, the ECF sigma factor SigV was already shown to have a pleiotrophic effect in the stress response of *E. faecalis* JH2-2 and was also suspected to be involved in its lysozyme resistance [26]. This constitutes a prominent concern since this antimicrobial component is considered as the first line of defense of the innate immune system, and also because *E. faecalis* is among the few bacteria that are completely lysozyme resistant.

**Figure 8:**
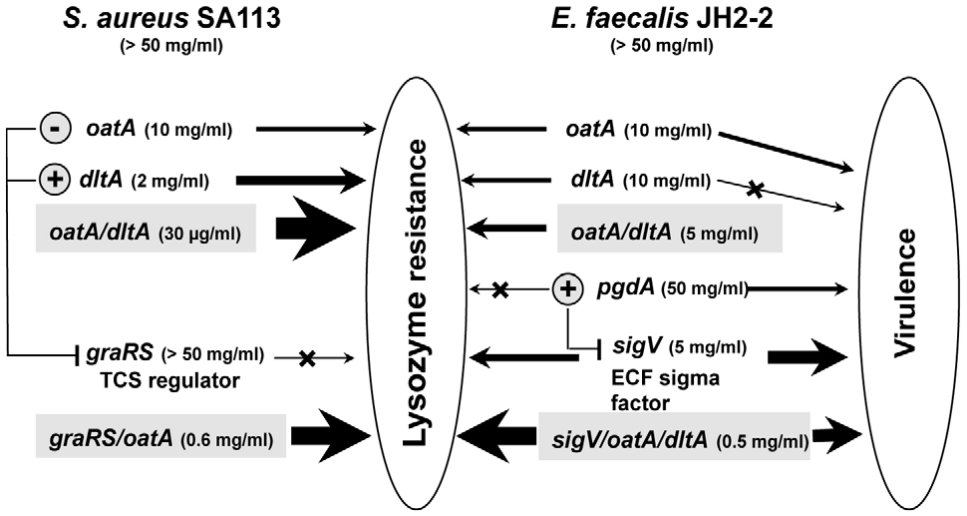
Comparison between the lysozyme resistance effectors of *S. aureus* and *E. faecalis*. The lowest concentrations of lysozyme resistance are indicated in parenthesis (data for *S. aureus* are from Herbert *et al*. [13]) and the most relevant are shaded. The relationship to virulence of the *E. faecalis* lysozyme resistance effectors is also indicated. Arrows thickness is relatively proportional to the involvement of the considered gene products in lysozyme resistance or virulence. Blocked arrows correspond to no effect on the considered event. Signs **+** and **–** correspond to up and down-regulation, respectively.

Lysozyme can affect bacteria by its muramidase and/or CAMP activities. To overcome the killing action of lysozyme, bacteria have developed different mechanisms among which some are well dissected. They are mainly based on the modification of the PG structure (*pgdA* and *oatA* for *S. pneumoniae* [14], [38]) associated to the reduction of the cell surface net charge (*oatA* and *dltA* for *S. aureus* [13], [15], or *pgdA*, *oatA* and *dlt* for *L. lactis* [39], [40]). However, the wild-type strains of the *E. faecalis* related lactic acid bacteria *S. pneumoniae* R36A [41] and *L. lactis* MG1363 [39], [40] have very low levels of lysozyme resistance (below 300 µg/ml). In terms of levels of lysozyme resistance, the most appropriate model for a comparative study is *S. aureus*, a human pathogen which also resists to high concentrations of lysozyme (50 mg/ml) [13], [15]. Thus, according to our results, the comparison of the lysozyme resistance mechanism models between *E. faecalis* and *S. aureus* was summarized in Fig. 8.

In *S. aureus*, the mechanism of the lysozyme resistance is based on muramidase (through *oatA* gene) and predominantly CAMP activities (through *dlt* operon). Indeed, the interruptions of both gene structures (*oatA/dltA* double mutant) sensitize the bacterium almost entirely to lysozyme (30 µg/ml) (Fig. 8) [13]. In addition, these gene structures are under the control of the glycopeptide resistance associated two-component system (TCS) regulator GraRS which regulates positively the *dlt* operon and negatively the *oatA* gene [13]. This is not the case for *E. faecalis* (Fig. 8).

The lysozyme resistance mechanism of *E. faecalis* is widely different from those reported above and obviously more complex as shown in Fig. 8. In *E. faecalis*, *oatA* but not *pgdA*-like was shown to contribute to lysozyme resistance [27]. Here we demonstrate that another part of lysozyme resistance (similar level to that conferred by *oatA* gene) is due to *dlt* genes. Since the *oatA*/*dltA* double mutant is twofold more sensitive to lysozyme than the respective single mutants, the contribution of each system seems to be additive. However, *oatA*/*dltA* double mutant still demonstrates a relatively high resistance to lysozyme (5 mg/ml), indicating that *oatA* and *dlt* genes can not be considered as the major determinants of *E. faecalis* lysozyme resistance. These findings revealed also that *E. faecalis* may certainly have additional effectors involved in its lysozyme resistance.

In this work, in depth investigation showed that *sigV* single mutant is as sensitive to lysozyme as *oatA*/*dltA* double mutant. In addition, the *sigV* deletion associated to that of *oatA* or *dltA* lead systematically to greater lysozyme sensitivity and the lowest level of resistance (0.5 to 1 mg/ml) was observed for *sigV/oatA*/*dltA* triple mutant. These results demonstrate a synergistic effect of *sigV*, *oatA*, and *dltA* genes, and confirm the key role of SigV in the lysozyme resistance of *E. faecalis*.

The transcriptional results suggest that lysozyme induces specifically *sigV* and its potential regulon members as it was shown for *pgdA*-like gene. Moreover, the overexpression of *sigV* either under lysozyme treatment or nisin induction has no incidence on the expression of *oatA* and *dltA* ruling out both genes from the *sigV* regulon. Our analyses showed also that the *sigV* mutant is not affected by the CAMP activity of nisin and thereby likely by that of lysozyme. Moreover, neither *dltA* nor *sigV* or *oatA* do contribute to the autolysis of *E. faecalis* JH2-2 under moderate detergent treatment.

To our knowledge, this is the first study demonstrating that an ECF sigma factor is the most important effector of lysozyme resistance in a bacterium. SigV as a transcriptional regulator does not control neither *oatA* nor *dlt* genes but it regulates positively the *pgdA*-like gene which was already shown to be not involved in the lysozyme resistance of *E. faecalis* [27]. Taken together our results argue in favor of a complex and original model of resistance to lysozyme of *E. faecalis* (Fig. 8). It is based at least on three effectors, *oatA*, *dltA* and mainly SigV ECF sigma factor through one or more gene(s) under its control, which remain to be identified.

Since lysozyme is considered as the first line of defense of the innate immune system, the bacterial resistance towards this immunogenic compound could be associated to virulence of pathogens. Regarding elucidated lysozyme resistance mechanisms; there are evidences in favor of this claiming. Indeed, *pgdA* of *S. pneumoniae* [41] and *dlt* genes of *S. pyogenes* [42], *S. suis* [43], *Listeria monocytogenes* [44] and *S. aureus* [45], [46] are shown to be involved in the virulence of these pathogens. It was already reported that MurNAc residues of the PG were *O*-acetylated only in pathogenic, lysozyme-resistant staphylococci allowing OatA to be regarded as a general virulence factor in *S. aureus* [47]. In association to PgdA, OatA also affects the fitness of *S. pneumoniae* [38] and deserves to retain our attention in the case of *E. faecalis*. Indeed, we have shown in a previous study a significant sensitivity in macrophages survival of the *oatA* mutant relatively to the wild-type strain [27]. In the present work, the recovery of this *oatA* mutant seems only slightly affected in the intravenous infection model in mice. This part of virulence could be related to lysozyme resistance as already hypothesized [27] but independently from SigV. Based on the assumptions described for intravenous [35] and urinary tract [48] infections, these observations suggest that OatA may contribute to the inflammatory response and to the persistence in mouse peritoneal macrophages but likely not in promoting colonization and adherence to uroepithelium. In contrast, there is no effect on virulence attenuation of the *E. faecalis dltA* mutant comparatively to the parental JH2-2 strain, at least in the models studied.

Many ECF sigma factors have been described to be involved in virulence of different bacteria such as *Mycobacterium tuberculosis*, *S. enterica* and *Pseudomonas aeruginosa* (for review, see [23], [24]). In this work, in addition to lysozyme resistance, the most striking role we have defined for SigV is its involvement in the virulence of *E. faecalis*. Using murine models of intravenous or UTI infections we demonstrated that in each of the tests applied, *E. faecalis* cells lacking a functional *sigV* gene were significantly impaired in their ability to establish and maintain infection for which a major hallmark is systemic dissemination, moving from the site of infection into the kidneys. In our analyses, the *sigV* and *sigV/oatA*/*dltA* mutants clearly showed a reduced capacity to colonize host tissues, as they are unambiguously less recovered from organs of mice subjected to intravenous or UTI infections than the wild-type JH2-2 strain. This demonstrates that SigV ECF sigma factor is certainly and more globally involved in virulence of *E. faecalis*. Interestingly, our results allowed also establishing through *oatA* and mainly *sigV* a direct link between lysozyme resistance and virulence of *E. faecalis*.

Unlike GraRS TCS regulator which controls the main effectors of the lysozyme resistance of *S. aureus*, SigV has no effect on *dlt* or *oatA* gene expressions (Fig. 8) arguing that hitherto unknown activities and mechanisms are responsible for this phenomenon in *E. faecalis*. On the other hand, the role of SigV could be paralleled to that of σ^S^, an ECF sigma factor of *S. aureus* recently described [49] as both are important components of the stress and pathogenic responses. In absence of a general stress response regulator in the genome sequence of *E. faecalis*, it appears that SigV can fulfill this role, at least in part, since it is not only involved in the response to harsh conditions [26] but also in the lysozyme resistance and virulence.

This work provides actual new insights that may help the investigation for new targets for antimicrobial drug development. The future purpose of our laboratory is to explore the mechanisms by which SigV constitutes an important regulatory component of the stress and pathogenic responses of *E. faecalis* in order to strengthen our knowledge about this potent opportunistic human pathogen.

## Materials and Methods

### Ethics statement

Murine work was performed under a protocol approved by the Institutional Animal Use and Care Committee at Università Cattolica del S. Cuore, Rome, Italy.

### Bacterial strains, plasmids, and culture conditions

Bacterial strains, plasmids, and oligonucleotide primers used in the present work are listed in Tables 3 and 4, respectively. *E. faecalis* JH2-2 strain and its derivatives were grown at 37°C without shaking, in M17 medium [50] supplemented with 0.5% glucose (GM17), or on otherwise specified media depending on requirements of the experiments. *E. coli* Top10F' strain was cultured with vigorous shaking at 37°C in LB broth. When required erythromycin (100 µg/ml) or ampicillin (100 µg/ml) was added.

**Table 3:** Bacterial strains and plasmids.

Strains and plasmids	Revelant characteristics	Reference or source
*E. faecalis* strains		
JH2-2	Fus^R^ Rif^R^, plasmid-free wild-type strain	[59]
* *Δ*sigV*	JH2-2 isogenic derivative EF3180 mutant (S26)	[26]
* *Δ*oatA*	JH2-2 isogenic derivative EF0783 mutant	[27]
* *Δ*dltA*	JH2-2 isogenic derivative EF2749 mutant	this study
* *Δ*sigV/*Δ*oatA*	JH2-2 isogenic derivative EF3180 and EF0783 double mutant	this study
* *Δ*sigV/*Δ*dltA*	JH2-2 isogenic derivative EF3180 and EF2749 double mutant	this study
* *Δ*oatA/*Δ*dltA*	JH2-2 isogenic derivative EF0783 and EF2749 double mutant	this study
* *Δ*sigV/*Δ*oatA/*Δ*dltA*	JH2-2 isogenic derivative EF3180, EF0783, and EF2749 triple mutant	this study
* SAS*	JH2-2 isogenic derivative *sigV-rsiV* double mutant	[26]
SAS pMSP3535	Em^R^, JH2-2 isogenic derivative *sigV-rsiV* double mutant with pMSP3535	[26]
SAS pMSP3535-*sigV*	Em^R^, JH2-2 isogenic derivative *sigV-rsiV* double mutant with pMSP3535-*sigV*	this study
* mprF*::pUCB300	Em^R^, JH2-2 isogenic derivative EF0031 mutant	this study
*E. coli* strain		
Top10F'	F' {lacIq Tn10 (TetR)} mcrA D(mrr-hsdRMS-mcrBC) f80lacZDM15 DlacX74 deoR recA1 araD139 D(ara-leu)7697 galU galK rpsL (StrR) endA1 nupG	Invitrogen
Plasmids		
pMAD	9.66 kb *ori* pE194^ts^, Em^R^ Amp^R^ *bgaB*	[54]
pMAD-Δ*sigV*	pMAD carrying *sigV* deletion	this study
pMAD-Δ*dltA*	pMAD carrying *dltA* deletion	this study
pUCB300	3.9 kb, *lacZ'* Amp^R^, Em^R^	[55]
pUCB300-*mprF*	pUCB300 carrying *mprF* internal fragment	this study
pMSP3535	8.35 kb Em^R^ nisR nisK P_nisA_ (nisin inductible promoter)	[56]
pMSP3535-*sigV*	pMSP3535 carrying *sigV* gene	this study

Ery, erythromycin; Rif, rifampin; Fus, fusidic acid; Amp, ampicillin; *bgaB*, β-galactosidase; nis, nisin.

R , resistant;

S , sensitive;

ts , thermosensitive.

**Table 4:** Oligonucleotide primers used in this study.

Primer name	Forward primer *	Reverse primer *	Operation
**dltA 5-6**	CGACAACTGaaTTCAGCTGTAAG (*Eco*RI)	ATTCTCCGTAgtCGACAATCGCTG (*Sal*I)	pMAD Cloning
**dltA 7-8**	GGAAAGTTAGTCGAcAACCTATTG (*Sal*I)	ACGATCGATGGAtCCTGAAGAAAT (*Bam*HI)	pMAD Cloning
**sigV 2V-FT3**	TCCGAGGAAtTCCTGCAAGGT (*Eco*RI)	GGATGGCCTgCAGTGCCTTTTC (*Pst*I)	pMAD Cloning
**sigV 3V-4V**	AGATGTACCtGCAGTTTGAATGA (*Pst*I)	CAATGACTaGATCtAGGAATCAC (*Bgl*II)	pMAD Cloning
**pMSP V1-2**	CGTCTATATAAGTcTgCAGGAAAGG (PstI)	GTTGGCGGCATgCTTTTGAAAAG (*Sph*I)	pMSP3535 Cloning
**Ef0031 2-3r**	GGTTCTGTATTAGaaTTCGTGGCTA (*Eco*RI)	GTTCCCATACCTGCAGGAACCATAG (*Pst*I)	pUCB300 Cloning
**dltA RT 1-2**	AACCAAGCTTCGATGGTGAA	AACGAGCGGCTAATTTCTCA	RT-qPCR
**LH 54-55 (** ***pgdA*** **)**	GCGATACCCAGGAGGTCATA	ACCATCGCATTCCAGTCAAT	RT-qPCR
**LH 56-57 (** ***oatA*** **)**	ACCTTCGTGAAAAAGGGACA	GTTGCGCACTTTTAGCGTTT	RT-qPCR
**SVRT 1-2 (** ***sigV*** ** )**	CGAAAGAAGATGCCTTGGAT	AAGAACCACGCGTCAAAATC	RT-qPCR

* Underlined nucleotides stand for the restriction sites (indicated in parenthesis) inserted within the primer sequences and nucleotides in lowercase are different from those of the target sequence.

### DNA manipulations

Restriction endonucleases and T4 ligase were obtained from Promega (Madison, Wi) and used in accordance with the manufacturer's instructions. Plasmids and PCR products were purified using Nucleospin plasmid and nucleospin extract II kits (Macherey-Nagel, Düren, Germany). Molecular cloning and other standard techniques were preformed essentially as previously described [51]. *E. coli* and *E. faecalis* strains were transformed by electroporation using Gene Pulseur Xcell (Bio-Rad Laboratories, Richmond, Ca, USA) as described by Dower *et al.,* [52] and Holo and Nes [53], respectively.

### Deletion mutagenesis

The construction of the JH2-2 derivative mutants was carried out with the pMAD plasmid by exploiting its property of thermo sensitive conditional replication [54]. Briefly, two fragments of approximately 900 bp corresponding to the flanking regions (including the 5′ and the 3′end parts, respectively) of the target genes were amplified by PCR using appropriate primers (Table 4). Then, they were purified, restricted with appropriate endonucleases and ligated into the pMAD vector in order to generate truncated allele of the gene of interest where the most part of the coding sequence (CDS) was deleted (60 to 80% of the CDS corresponding to the median part of the gene). The ligation mixture was transformed by electroporation into *E. coli* Top10F' cells. After selection and verification, the generated recombinant plasmids were used to transform *E. faecalis* JH2-2 electrocompetent cells and gene replacement was performed via double cross over events as described previously [27].

### Construction of the *mprF* insertional mutant

The *mprF* insertionnal mutant was constructed following another procedure. Briefly, an internal fragment of *mprF* gene was amplified by PCR with specific primers (Table 4), digested by *EcoR*I and *Pst*I and ligated in the suicide vector pUCB300 [55] previously treated with the same enzymes. The ligation product was electroporated into *E. coli* Top10F' cells. The subsequent recombinant plasmid (pUCB300-*mprF*) (Table 3) was transformed in *E. faecalis* JH2-2. The generated derivative mutant (*mprF*::pUCB300) (Table 3) was verified by PCR for the insertion of the recombinant plasmid pUCB300-*mprF* within the chromosome leading to inactivated *mprF* gene.

### Lysozyme sensitivity

Lysozyme sensitivity assays were performed on LB medium plates containing different concentrations (0 to 20 mg/ml) of hen egg white lysozyme (HEWL) (Fluka, Buchs, Switzerland). Overnight cultures of the parental JH2-2 strain and its derivative mutants were adjusted to OD_600_ of 1 in physiological water, and diluted up to 10^−3^. An equal volume (5 µl) of the 10^−1^, 10^−2^, and 10^−3^ dilutions was then spotted on LB plates with, or without lysozyme. The bacterial growth was evaluated after 48 hours of incubation at 37°C and photographed.

### Nisin sensitivity

In order to assess the effect of nisin on the kinetic growth of *E. faecalis* JH2-2 and its derivative mutants, strains were grown on GM17 to mid-log phase. At this step, 40 ml of fresh medium was inoculated in order to start the culture from OD_600_ of 0.05 and divided into two parts. The first 20 ml culture was carried out without supplementation (control) and the second received 2 µg/ml of nisin (Sigma Chemical Co, St. Louis, Mo, USA). The growth kinetic of the two cultures was then monitored at OD_600_ nm during 8 hours.

### Triton X-100-induced autolysis assays under non-growing conditions

Strains were grown to an OD_600_ of 0.8 in GM17 medium and treated as previously described [34]. Briefly, cells were harvested by centrifugation (4000 g, 10 min at 4°C), washed with ice-cold sterile water, and then resuspended in the same volume of 50 mM Tris-HCl, pH 7.5, containing 0.05% or 0.1% Triton X-100. The cell suspensions were then transferred into 100-well sterile micro plates and incubated at 37°C without shaking. Autolysis was monitored by measuring OD_600_ of the cell suspensions every 30 min, with an automated incubator/optical density reader (Model 680, Bio-Rad Laboratories).

### Construction of SAS pMSP3535-*sigV* strain

In order to overproduce the ECF sigma factor SigV, we cloned its corresponding gene into pMSP3535 plasmid [56]. Briefly, the entire gene with its own translation signal was amplified by PCR using the appropriate primers (Table 4) and then inserted under the control of the nisin inducible promoter (*PnisA*) of pMSP3535 plasmid. The subsequent recombinant plasmid (pMSP3535-*sigV*) (Table 3) previously obtained in *E. coli* TOP10F' was transformed into electro-competent *E. faecalis* JH2-2 SAS cells generating the SAS pMSP3535-*sigV* strain (Table 3) which overproduces the SigV sigma factor.

### RNA isolation and RT-qPCR experiments

In order to assess comparative transcriptional gene expression, we used JH2-2 wild-type strain and its *sigV* derivative mutant cultured on GM17 medium supplemented with 3 mg/ml lysozyme. On the other hand, to analyze and verify the effect of SigV overproduction on *oatA*, *pgdA*, *dltA* and on its own *sigV* gene expression, SAS pMSP3535 and SAS pMSP3535-*sigV* strains (Table 3) were used under nisin induction. Using RNeasy Midi Kit (Qiagen, Valencia, Ca, USA), three or two independent samples of total RNA were isolated for each condition, respectively. Prior to the extraction, the mid-log phase (OD_600_ = 0.4) cultures were treated during 30 minutes with 3 mg/ml lysozyme or 0.5 µg/ml of nisin.

For reverse transcriptase-quantitative real-time PCR (RT-qPCR), specific primers were designed using the *E. faecalis* V583 genome sequence and the Primer3 software (http://frodo.wi.mit.edu/cgi-bin/primer3/primer3_www.cgi). Primer pairs listed in Table 4 were designed in order to produce amplicons of equivalent length (100 bp). Two micrograms (2 µg) of total RNA were reverse transcribed with random hexamer primers and Omniscript enzyme (Qiagen). Quantification of 23S rRNA or *gyrA* (encoding the gyrase enzyme) levels was used as an internal control. Amplification (carried out with 5µl of cDNA dilution 1/100), detection (with automatic calculation of the threshold value), and real-time analysis were performed twice with cDNA samples using the iCycler iQ detection system (Bio-Rad Laboratories). The relative mRNA expression level of each gene in each sample was calculated using comparative cycle time as described previously [57].

### Animal studies

Animal experiments were performed with the approval of the Institutional Animal Use and Care Committee at Università Cattolica del S. Cuore, Rome, Italy. Female BALB/c mice, 20 to 25 g, (Harlan Italy S.r.l) were housed in filter-top cages with free access to food and water at the Catholic University Unit for Laboratory Animal Medicine. In order to assess the virulence of the *oat*, *dltA*, *sigV* single-mutant strains and of the *oat*/*dltA*/*sigV* triple mutant strain with respect to the JH2-2 wild-type strain, two different mouse models were used.

In the intravenous infection model, experiments were performed according to Gentry-Weeks *et al.*, [35]. Briefly, overnight cultures of the strains grown in brain-hearth infusion broth (BHI) supplemented with 40% heat-inactivated horse serum were centrifuged and the resulting pellets were resuspended in sterile PBS to achieve final concentrations of 1×10^9^ bacteria/ml. Aliquots of 100 µl from each strain suspension were used to inject groups of 10 mice into their tail veins. Infections experiments were repeated three times. The mice were monitored with twice-daily inspections and they were sacrificed using CO_2_ inhalation at 7 days after infection. Kidneys and livers were then removed aseptically, weighed, and homogenized in 5 ml of PBS using a Stomacher 80 (Pbi International, Milan, Italy) for 120 s at high speed. Serial homogenate dilutions were plated onto *Enterococcus* selective agar (Fluka Analytical, Switzerland) for CFU determination.

In the urinary tract infection (UTI) model, we followed a previously described protocol [36]. Briefly, each bacterial strain was grown in 10 ml of BHI broth supplemented with 40% heat-inactivated horse serum for 10 h at 37°C under shaking. Cells were pelleted, resuspended in 10 ml of sterile PBS, and then adjusted to reach a concentration of 1×10^7^ cells/ml. First, groups of five isoflurane-anesthetized mice per bacterial inoculum (10^2^ to 10^6^ CFU) were infected via intraurethral catheterization (polyethylene catheter, ∼4 cm long; outer diameter, 0.61 mm; Becton Dickinson, Sparks, MD) with 200 µl of each strain suspension. Additionally, groups of 15 mice were infected with a sole inoculum of 10^4^ CFU. Mice were sacrificed 48 h after transurethral challenge, bladders and kidney pairs were processed as described above. For each strain, the 50% infective dose (ID_50_) was determined as previously described [58]. The bacterial detection limits were 50 CFU/ml for kidneys and 10 CFU/ml for bladder homogenates. Differences between the total numbers of infected kidney pairs or bladders, obtained by combining all inoculum (10^2^ to 10^6^ CFU) groups were analyzed by Fisher's exact test.

For both models, CFU counts were analyzed by unpaired *t* test. For all comparisons, a *p* value of less than 0.05 was considered to be significant.
